# Trends in the use of emergency contraception in Britain: evidence from the second and third National Surveys of Sexual Attitudes and Lifestyles

**DOI:** 10.1111/1471-0528.14131

**Published:** 2016-05-31

**Authors:** KI Black, R Geary, R French, N Leefe, CH Mercer, A Glasier, W Macdowall, L Gibson, J Datta, M Palmer, K Wellings

**Affiliations:** ^1^ University of SydneySydneyNSWAustralia; ^2^ London School of Hygiene and Tropical MedicineLondonUK; ^3^ University College LondonLondonUK; ^4^ University of EdinburghEdinburghUK

**Keywords:** Emergency contraception, pharmacy access, risk factors, sexual behaviour, unplanned pregnancy

## Abstract

**Objective:**

To examine the changes in the prevalence of, and the factors associated with, the use of emergency contraception (EC) in Britain between 2000 and 2010, spanning the period of deregulation and increase in pharmacy supply.

**Design:**

Cross‐sectional probability sample surveys.

**Setting and population:**

British general population.

**Methods:**

Data were analysed from the second and third British National Surveys of Sexual Attitudes and Lifestyles (Natsal), undertaken in 1999–2001 and 2010–12. Univariate and logistic regression analyses were used to measure change in EC use amongst sexually active women aged 16–44 years not intending pregnancy.

**Main outcome measures:**

Prevalence of EC use and factors associated with use.

**Results:**

Of the 5430 women surveyed in 1999–2001 and the 4825 women surveyed in 2010–12, 2.3 and 3.6%, respectively, reported using EC in the year prior to interview (*P *= 0.0019 for change over time). The prevalence of EC use increased amongst single women and those with higher educational attainment (adjusted odds ratio, aOR 1.51; 95% confidence interval, 95% CI 1.04–2.20; *P *= 0.0308). Increases in EC use were generally greater among women without behavioural risk factors, such as those with no history of abortion within 5 years (aOR 1.57; 95% CI 1.17–2.12; *P *= 0.0029), or those whose first heterosexual intercourse occurred after the age of 16 years (aOR 1.68; 95% CI 1.21–2.35; *P *= 0.0021). The increase in EC use was also more marked among women usually accessing contraception from retail sources than among those doing so from healthcare sources, which may reflect a use of condoms amongst EC users.

**Conclusion:**

The increase in EC use among women in Britain in the first decade of the 21st century was associated with some, but not all, risk factors for unplanned pregnancy. Advice and provision may need to be targeted at those at highest risk of unplanned pregnancy.

**Tweetable abstract:**

Despite pharmacy access, only a small rise in emergency contraception use has been seen in Britain over 10 years.

## Tweetable abstract

Despite pharmacy access, only a small rise in emergency contraception use has been seen in Britain over 10 years.

## Introduction

Emergency contraception (EC), including oral and intrauterine methods, occupies a unique position amongst contraceptive methods, in that it can be used after sex to prevent pregnancy.^1^ EC use has been promoted as a component of strategies to reduce unintended pregnancy.^2^, ^3^ EC use can be considered a marker of risky sexual behaviour, as it indicates exposure to unprotected sex or a failure in contraceptive method. Although the impact of EC use on unintended pregnancy rates at a population level has not yet been established,^4^ at the individual level, for women seeking to avert an unintended pregnancy after unprotected intercourse, trial data indicate that intrauterine devices will prevent 99% of pregnancies,^5^ and that oral EC prevents around two‐thirds of pregnancies if commenced within 24 hours.^6^, ^7^, ^8^

Access to oral EC has changed dramatically in the UK in the last 15 years.^9^ Major changes were facilitated by the development of a progestogen‐only formulation containing levonorgestrel, which is both safe to use and has no significant contraindications. As a consequence, the levonorgestrel formulation was deregulated and became available over the counter (OTC) from pharmacies without prescription in the UK in 2001, but prior to that some National Health Service (NHS) areas had already enabled pharmacy access via a Patient Group Direction. Furthermore, in 2009, ulipristal acetate, a selective progesterone receptor modulator, was licensed for EC use with efficacy demonstrated up to 120 hours after unprotected sexual intercourse or contraceptive failure.^10^ It became accessible only on prescription in the UK until April 2015 when the European Medicines Agency reviewed the safety data and changed its status to an OTC medication.^11^ Emergency copper intrauterine devices continue to be available free at the point of access through community family planning and general practice clinics, and although significantly more reliable compared with pills,^5^ are less accessible as they require insertion by a healthcare practitioner, with same‐day insertion being an additional challenge. In tandem with these advances, public education advertisements have informed women about the role of EC after unprotected sex,^12^, ^13^ but, at least in the first few years of the OTC availability of levonorgestrel EC, the improved information and access did not translate into increased uptake.^14^

Data from the second and third British National Surveys of Sexual Attitudes and Lifestyles (Natsal) permit an examination of sociodemographic and sexual lifestyle factors associated with the reported use of EC among women resident in Britain. The two surveys were carried out in 1999–2001 and 2010–12, respectively, before and after the deregulation of oral EC in Britain. In this paper we report a change in the prevalence of use of EC between the surveys, together with factors associated with use and their differences between the surveys.

## Methods

To date, three Natsal probability sample surveys have been carried out, approximately decennially: in 1990–1991 (Natsal‐1), in 1999–2001 (Natsal‐2), and in 2010–12 (Natsal‐3). In this paper we used data from Natsal‐2 and Natsal‐3 to examine trends in use of EC over time before and after the deregulation of oral EC in Britain. In Natsal‐2, 11 161 respondents (6399 women aged 16–44 years) were interviewed, and participants resident in London were oversampled. Natsal‐3 interviewed 15 162 men and women aged 16–74 years (5842 women aged 16–44 years), with an oversampling of women aged 16–34 years to allow for a detailed exploration of behaviours in the age group at highest risk of sexual health outcomes such as unplanned pregnancy. The unadjusted response rate in Natsal‐2 was 63.1% and the adjusted rate, taking account of over‐sampling in London, was 65.4%. The response rate for Natsal‐3 was 57.7% and the cooperation rate was 65.8% (of the eligible addresses contacted).

Detailed descriptions of the methodology for Natsal‐2 and Natsal‐3 have been published elsewhere.^15^, ^16^, ^17^ In all three Natsal surveys, households were selected using stratified probability sampling, from which one eligible individual, resident in Britain (England, Scotland, or Wales), was selected at random and invited to participate. Participants were interviewed in their own homes through a combination of face‐to‐face computer‐assisted personal interviews (CAPI) and computer‐assisted self‐interview (CASI), for the more sensitive questions.

As in Natsal‐2, in Natsal‐3 we weighted the data to adjust for the unequal probabilities of selection in terms of age and the number of adults in the eligible age range at an address. After application of these selection weights, the sample was broadly representative of the British population compared with 2011 census figures, although men and London residents were slightly under‐represented.^17^ Therefore, we also applied a non‐response post‐stratification weighting to correct for differences in gender, age, and Government Office Region between the achieved sample and the 2011 census.^17^, ^18^, ^19^ Natsal‐2 was approved by the research ethics committees of University College London and the North Thames Multicentre (3 June 1998) and by all of the local research ethics committees in Britain. The Natsal‐3 study was approved by the Oxfordshire research ethics committee A (reference: 09/H0604/27) on 12 July 2010. Participants provided oral informed consent for interviews.

### Measures

In the CAPI component of the questionnaire, participants who reported ever using any contraceptive method(s) were asked which, if any, and including EC, they had used with a partner in the past year. The wording of the question enabled women to report using EC in Natsal‐2 and using the ‘morning‐after pill’ and/or an emergency intrauterine device (IUD) in Natsal‐3. For comparability with Natsal‐2, Natsal‐3 responses for the morning‐after pill and emergency IUD were combined to create a measure of overall EC use in the past year. Other contraceptive method(s) used in the past year were classified according to the most effective method reported. Methods with a typical‐use failure rate (which includes incorrect and inconsistent use) below 10% were classified as more effective (IUD, intrauterine system, implant, injection, patch, and oral contraceptive pill).^20^ Those with a typical‐use failure rate of more than 10% were classified as less effective [condoms (male and female), diaphragm, pessaries, gels, emergency contraception, withdrawal, rhythm method, and no method].^20^ Participants using any contraceptive method(s) in the past year were shown a card listing different sources of supplies and asked to indicate which source(s) they had used in the past year. Participants could report multiple sources. In analyses, sources accessed to obtain contraception were grouped as clinical [doctor or nurse in general practice, genitourinary medicine (GUM)/family planning/contraceptive or reproductive health clinic, or youth services] or retail/other (pharmacy/chemist, website, petrol station/supermarket/other shop, vending machine, or mail order and other).

The information from the CASI questions was used to calculate any sexually transmitted infection (STI) diagnosis (chlamydia, gonorrhoea, syphilis, herpes, genital warts, trichomonas, or non‐gonococcal/non‐specific urethritis) in the past year. Ethnic origin was derived from the Office for National Statistics harmonised question on ethnicity. The index of multiple deprivation (IMD) was used as an area‐level measure of social status.^21^Educational attainment was defined according to school leaving age and academic qualifications obtained (individual level). Religiosity was derived from self‐reported current importance of religion (important or very important) and frequency of attendance at religious services or meetings (at least twice a year). Average weekly alcohol consumption was derived from average frequency of consumption in the last year and average intake when drinking (excluding special occasions). Gender‐specific limits (>21 units for men and >14 units for women) were used to define exceeding the recommended average consumption.^22^

### Statistical analyses

We used stata 13.1 for complex survey analysis to incorporate the weighting, clustering, and stratification of the Natsal data. We present descriptive statistics of the prevalence of use of EC in 1999–2001 and 2010–12 among heterosexually active women aged 16–44 years (defined as those reporting at least one partner of the opposite gender in the past year). We used logistic regression to examine change in the prevalence of EC use between 1999–2001 and 2010–12 by key sociodemographic characteristics, average alcohol consumption, and key sexual behaviours. We included interaction terms to test whether the magnitude of change in EC use between the two surveys differed by key sociodemographic characteristics, average alcohol consumption, and key sexual behaviours. All regression analyses were adjusted for age. We used an *α* value of 0.05 in all analyses.

## Results

Of the 6399 women interviewed for Natsal‐2, 5462 (87%) women reported at least one male partner in the past year, of whom 5430 had complete data on EC use. The number of women interviewed and included in the denominator varies slightly from that reported in a previous publication of the profile of EC users from Natsal‐2 (Black et al. *Contraception* 2006;74:309–312), as this analysis is restricted to participants included in the core Natsal‐2 sample, and does not include the Natsal‐2 ethnic minority boost. This has not influenced the estimate of the prevalence of EC use in the past year for 1999–2001. Of the 5842 women aged 16–44 years interviewed for Natsal‐3, 4889 (85%) reported at least one male sexual partner in the past year, of whom 4825 had complete data on EC use.

In 2010–12, 3.6% of women reported having used EC in the past year, compared with 2.3% in 1999–2001 (Table 1). The increase between the two time periods was statistically significant (*P *= 0.0019). There was a strong age gradient in both 1999–2001 and 2010–12, with use among 16–24 year olds being considerably higher than among women aged 40–44 years.

**Table 1:** Use of EC in past year among sexually active women aged 16–44 years by demographic characteristics: 1999–2001 and 2010–2012

	1999–2001			2010–2012			Age‐adjusted OR	95% CI	*P*
	Denominators (unweighted, weighted)	Percentage	(95% CI)	Denominators (unweighted, weighted)	Percentage	(95% CI)			
**Total**	5430, 4859	2.30	(1.9–2.8)	4825, 3375	3.60	(3.0–4.3)	1.54	(1.17–2.02)	0.0019
**Age group**									
16–24 years	1110, 1158	5.20	(4.0–6.9)	1604, 888	7.10	(5.7–8.9)	1.39	(0.95–2.03)	0.0887
25–29 years	1064, 870	2.40	(1.7–3.6)	1257, 624	5.10	(3.7–7.1)	2.16	(1.29–3.61)	0.0035
30–39 years	2324, 1988	1.50	(1.0–2.2)	1460, 1204	1.60	(1.1–2.3)	1.08	(0.62–1.88)	0.7907
40–44 years^a^	932, 843	0.10	(0.0–0.5)	504, 659	1.10	(0.5–2.5)	8.10	(1.57–41.75)	0.0124
**Ethnic origin**									
White	4897, 4501	2.20	(1.8–2.7)	4245, 2929	3.30	(2.7–4.0)	1.52	(1.14–2.02)	0.0040
Asian/Asian British	144, 127	6.50	(2.6–15.1)	230, 194	3.30	(1.7–6.3)	0.49	(0.15–1.57)	0.2311
Black/Black British	209, 109	2.80	(0.8–9.0)	148, 117	8.30	(4.4–15.3)	3.20	(0.78–13.18)	0.1057
Other^b^	169, 112	2.30	(0.8–6.6)	157, 106	8.00	(4.2–14.5)	3.71	(1.03–13.43)	0.0445
**Relationship status**									
Married/cohabiting	3345, 3351	1.20	(0.9–1.7)	2381, 2060	1.10	(0.8–1.6)	0.98	(0.59–1.62)	0.9393
Previously/never married	2079, 1503	4.70	(3.7–5.9)	2433, 1308	7.50	(6.2–9.1)	1.64	(1.19–2.26)	0.0024
**Religiosity** c									
No	856, 795	2.00	(1.2–3.6)	527, 369	2.80	(1.6–4.8)	1.27	(0.53–3.04)	0.5943
Yes	1074, 892	1.90	(1.2–3.2)	892, 716	3.80	(2.4–6.0)	1.82	(0.91–3.64)	0.0913
**Academic qualifications** d									
No academic qualifications	829, 714	1.00	(0.5–2.2)	401, 259	1.80	(0.8–3.7)	1.54	(0.52–4.53)	0.4346
Academic qualifications typically gained at age 16^e^	2396, 2185	1.90	(1.4–2.6)	1626, 1141	2.30	(1.7–3.1)	1.29	(0.82–2.02)	0.2703
Studying for/attained further academic qualifications	2043, 1800	3.30	(2.4–4.4)	2545, 1810	4.80	(3.9–6.0)	1.51	(1.04–2.20)	0.0308
**Index of multiple deprivation** f									
(least deprived)	792, 741	1.80	(1.0–3.2)	797, 596	3.30	(2.2–4.8)	1.82	(0.88–3.74)	0.1045
2	817, 788	3.10	(2.0–4.7)	860, 642	3.30	(2.3–4.9)	0.98	(0.53–1.82)	0.9570
3	880, 856	2.90	(1.8–4.7)	948, 676	3.10	(2.2–4.4)	0.99	(0.54–1.81)	0.9683
4	1230, 1123	2.50	(1.6–3.7)	1071, 744	4.40	(3.0–6.5)	1.83	(1.03–3.27)	0.0399
(most deprived)	1711, 1351	1.70	(1.1–2.6)	1149, 718	3.70	(2.7–5.2)	2.26	(1.29–3.96)	0.0045

a Significant age group/survey interaction, indicating that the change has been significantly different among those age 40–44 years relative to those aged 16–24 years.

b Combines those reporting mixed, Chinese, or other ethnic origins because of the small number of participants reporting these ethnic origins.

c Religiosity was derived from self‐reported importance of religion and religious beliefs now, and frequency of attendance at religious services or meetings. Religiosity was defined as reporting that religion was very important or fairly important, with attendance at religious services or meetings at least twice a year.

d Participants aged ≥17 years.

e English General Certificate of Secondary Education or equivalent.

f Index of Multiple Deprivation (IMD) is a multi‐dimensional measure of area (neighbourhood)‐level deprivation based on the participant's postcode. IMD scores for England, Scotland, and Wales were adjusted before being combined and assigned to quintiles, using a method by Payne and Abel.

In both 1999–2001 and 2010–12, EC use was more commonly reported by those previously or never married than by those married or cohabiting. Use increased significantly over the period among those previously or never married (*P *= 0.0024), but not among married or cohabiting women. There was no consistent variation in EC use by area‐related deprivation level; however, an increase in use between the two time periods was seen for women living in areas in the two most deprived quintiles of area‐related deprivation, but not among women from other socio‐economic areas.

The use of EC was more commonly reported among women studying for, or having attained, educational qualifications, beyond those minimally gained at age 16 years, in both time periods, but this difference was only significant in 2010–12. Furthermore, it was only in the highest category of educational attainment that there was a significant increase in use over the period. With regards to ethnicity, the proportion of EC users in 1999–2001 was highest among women self‐identifying as Asian, whereas in 2010–12 it was highest among women self‐identifying as black or ‘other’ (mixed, Chinese, or other). Age‐adjusted odds ratios (aORs) for increase in use were only significantly raised over time for women in the ‘white’ (aOR 1.52; 95% CI 1.14–2.02; *P *= 0.0040) and ‘other’ (aOR 3.71; 95% CI 1.03–13.43; *P *= 0.0445) ethnic groups, but small numbers in some ethnic categories caution against over‐interpretation.

In terms of sexual risk behaviour, the prevalence of EC use was higher among women who reported abortion within the last 5 years, among women with more than one sexual partner in the past year, and among women who attended a sexual health clinic in the past year for both time periods (Table 2). It was significantly associated with both STI diagnosis in the past year and higher than recommended alcohol consumption in 2010–12, but not in 1999–2001. In neither time frame was EC significantly associated with intercourse before the age of 16 years. The confidence intervals suggest that the association with the use of a less reliable or no contraception was not significant in 1999–2001, and barely reached significance in 2010–12 (Table 2).

**Table 2:** Use of EC in past year among sexually active women aged 16–44 years by behavioural characteristics: 1999–2001 and 2010–2012

	1999–2001			2010–2012			Age‐adjusted OR for change over time	(95% CI)	*P*
	Denominators	Percentage	(95% CI)	Denominators	Percentage	(95% CI)			
**Total**	5430, 4859	2.30	(1.9–2.8)	4825, 3375	3.60	(3.0–4.3)	1.54	(1.17–2.02)	0.0019
**Heterosexual intercourse before age 16 years**									
No	4257, 3792	2.00	(1.6–2.6)	3347, 2493	3.40	(2.7–4.2)	1.68	(1.21–2.35)	0.0021
Yes	1172, 1066	3.40	(2.3–4.9)	1478, 882	4.30	(3.3–5.6)	1.29	(0.80–2.08)	0.2968
**Abortion in the past 5 years**									
No	5024, 4544	2.10	(1.7–2.6)	4489, 3196	3.40	(2.8–4.1)	1.57	(1.17–2.12)	0.0029
Yes	398, 311	5.30	(3.2–8.5)	320, 170	7.90	(5.2–11.8)	1.53	(0.78–2.98)	0.2141
**Number of sexual partners in the past year**									
1	4468, 4093	1.70	(1.4–2.3)	3731, 2756	1.90	(1.6–2.4)	1.10	(0.78–1.57)	0.5761
2 or more^a^	927, 738	5.50	(4.0–7.6)	1092, 619	11.00	(8.6–13.9)	2.09	(1.36–3.23)	0.0009
**Diagnosed with an STI in the past year** b									
No	5119, 4615	2.30	(1.9–2.8)	4694, 3303	3.40	(2.9–4.0)	1.49	(1.13–1.96)	0.0050
Yes	86, 73	3.50	(0.9–11.8)	103, 56	14.90	(7.0–28.7)	4.56	(0.95–22.03)	0.0568
**Attended a sexual health clinic in the past year**									
No	5273, 4746	2.20	(1.8–2.7)	4209, 3025	2.80	(2.3–3.4)	1.33	(0.99–1.78)	0.0579
Yes	152, 110	6.90	(3.6–12.9)	565, 319	11.70	(8.5–15.8)	1.68	(0.75–3.76)	0.2045
**Usual contraceptive method, past year** c									
Reliable method	3018, 2770	2.10	(1.6–2.8)	2808, 1902	2.90	(2.3–3.7)	1.31	(0.90–1.91)	0.1577
Less reliable or no method	2207, 1911	2.30	(1.7–3.2)	2011, 1469	4.40	(3.4–5.6)	1.93	(1.29–2.88)	0.0013
**Source of contraceptive supplies, past year**									
Not got contraceptive supplies	1873, 1709	0.20	(0.1–0.6)	655, 594	1.10	(0.5–2.1)	4.16	(1.22–14.21)	0.0229
Clinical	3052, 2702	3.90	(3.2–4.8)	2967, 1862	4.90	(4.0–6.0)	1.29	(0.96–1.74)	0.0907
Retail/Other	505, 448	0.80	(0.3–2.2)	505, 390	6.00	(4.2–8.5)	8.43	(2.76–25.78)	0.0002
**Average alcohol consumption per week**									
None/Not more than recommended	4865, 4361	2.10	(1.7–2.6)	4196, 2959	3.10	(2.6–3.8)	1.44	(1.08–1.93)	0.0139
More than recommended	563, 496	3.90	(2.3–6.5)	607, 404	7.00	(4.6–10.5)	1.89	(0.94–3.79)	0.0724

a Significant number of sexual partners/survey interaction indicating that the change has been significantly different among those reporting two or more sexual partners in the past year, relative to those reporting.

b Chlamydia, gonorrhoea, syphilis, herpes, genital warts, trichomonas, or non‐gonococcal urethritis.

c Reported usual method of contraception used in the past year was classified according the most effective method reported. Methods with a typical use failure rate (including incorrect and inconsistent use) below 10% were classified as more effective [IUD, intrauterine systems (IUS), implant, injection, patch, and oral contraceptive pill]. Those with a typical use failure rate of more than 10% were classified as less effective [condoms (male and female), diaphragm, pessaries, gels, EC, withdrawal, rhythm method, and no method].

The increase in use of EC between the two time periods was associated appreciably with some, but not all, risk factors for unplanned pregnancy. A significant increase in use was seen in 2010–12 among women with two or more sexual partners in the past year (aOR 2.09; 95% CI 1.36–3.23; *P *= 0.0009). A significant increase in use was also seen in the more recent time period among women using a less reliable method of contraception or none (aOR 1.93; 95% CI 1.29–2.88; *P *= 0.0013; Table 2). For other risk factors, however, such as abortion in the last 5 years or heterosexual sex before the age of 16 years, the increase in the use of EC was greater among women who did not report these, than among those who did. EC use increased significantly between the surveys among women whose first intercourse occurred at age 16 years or later, but not among women who were sexually active before the age of 16 years; it increased significantly among women who had no experience of abortion in the past 5 years, but not among women who did; and an increase was seen among women who were not diagnosed with an STI in the past year, but not among those who were.

In terms of health service attendance, there was higher prevalence of EC use among women who usually sourced their contraceptive supplies from clinical services compared with retail sources in 1999–2001. This pattern was reversed in 2010–12, when the proportion of women using EC in the past year was higher among women using retail sources compared with women using clinical sources (0.8 versus 3.9% in 1999–2001; 6.0 versus 4.9% in 2010–12). Age‐adjusted odds show that EC use increased between the two time periods among users of retail sources of contraception (aOR 8.43; 95% CI 2.76–25.78; *P *= 0.0002), whereas the increase among users of clinical services barely reached significance (aOR 1.29; 95% CI 0.96–1.74; *P *= 0.0579).

## Discussion

### Main findings

These data from two serially conducted national probability sample surveys show a small but significant increase in EC use among heterosexually active women aged 16–44 years in Britain in the first decade of the 21st century. Our data show that that this increase was considerably greater among women routinely using retail outlets for contraceptive supplies than among women using clinical services. The increase in use of EC has also been greater among women who live in less affluent areas and also among those who are currently single. In terms of risk factors for unplanned pregnancy, the picture is more mixed. We saw a sizeable increase in the use of EC in the last survey among women using less effective methods of contraception, such that EC use is now more common among this group than among women with higher levels of contraceptive protection. At the same time, although prevalence of EC use was higher among women who had an abortion in the preceding 5‐year period, the increase in use over time was larger among those who had not undergone abortion. Furthermore, associations with EC use are generally stronger, and the increase in prevalence is generally greater, for indicators of STI risk (multiple sexual partners and STI diagnosis) than for indicators of risk of unplanned pregnancy (abortion and earlier sexual experience).

### Strengths and limitations

This large‐scale, population‐based study has advantages over the use of routine data in measuring changes in patterns of EC use over time, notably our ability to describe the changing characteristics of EC users. Nevertheless, even in a sample of this size, the relative rarity of some experiences – an STI diagnosis, for example – limit the extent to which we are able to detect significant associations. A further limitation results from the cross‐sectional nature of the study, such that causal direction cannot be established. We cannot know, for example, whether the start of use of the current contraceptive method preceded EC use or was subsequent to it, and this hampers interpretation.

### Interpretation

One of the most striking findings of the study, an eight‐fold increase in EC use between the time periods among women using retail sources to obtain contraception, is confirmed in routinely collected prescription NHS data. EC prescriptions dispensed from sexual and reproductive health services as well as clinical community sources (predominantly general practitioners) has been falling during the period under study, such that in 2012–13 the number of prescriptions for oral EC was less than half that in 2000–01.^23^ Furthermore, the Office of National Statistics reported in 2003–04 that 27% of women obtained their EC from community pharmacies, and by 2007–08 this had risen to 51%.^24^, ^25^ These data point to a shift to over‐the‐counter access coinciding with the deregulation of EC supply in Britain. A study spanning the period of pharmacy deregulation in France reported that, by 2004, most women (60.1%) stated that the last time that they used oral EC they obtained it directly from the pharmacy without a prescription.^26^

Although the increase in use of EC apparently coinciding with deregulation is to be welcomed, our data do not fully support the view that the concomitant increase in uptake has been greatest among those at highest risk of unplanned pregnancy. Studies have highlighted several potential limitations of pharmacy supply compared with access to clinical services. This suggests that barriers for access to EC remain for some women, possibly related to a lack of knowledge about EC and/or a lack of ease in requesting the medication.^27^

Effective targeting of women most at risk of unplanned pregnancy is one challenge to be overcome in the pharmacy supply of EC. There is some evidence of deficiencies in the provision of information about contraceptive methods at the time of obtaining EC in pharmacies,^28^, ^29^ although larger, population‐based studies have not shown an adverse effect on contraceptive uptake of greater EC access through pharmacies. Women obtaining EC through pharmacies in France, for example, were no less likely to use more effective methods of contraception than women obtaining it from clinical sources.^26^ Nevertheless, the pharmacy encounter is a potential opportunity to provide information about contraception, and in a recent pilot study of pharmacy provision of oral contraception in South London, the pharmacy supplying the highest number of pill prescriptions also saw a significant fall in requests for EC, although this finding was not consistent across all outlets.^29^

## Conclusion

This study has provided information on changing patterns of EC use. The increased prevalence of use among women with some risk factors, but not all, suggests that despite deregulation, barriers to access remain. We highlight the importance of strategies to ensure that women are provided with information at the point of supply that allows them to make informed choices about their continuing contraceptive needs.

## Disclosure of interests

Full disclosure of interests available to view online as supporting information.

## Contribution to authorship

KIB, KW, and CHM conceived this article. KIB and KW wrote the first draft of the article, with further contributions from JD, RF, RG, LG, AG, WM, MP, and CHM. RG and NL performed the statistical analysis, with support from CHM. KW, CHM, and WM, initial applicants for Natsal‐3, wrote the study protocol and obtained funding. KW, WM, JD, and CHM designed the Natsal‐3 questionnaire, applied for ethics approval, and undertook piloting of the questionnaire. All authors interpreted data, reviewed successive drafts, and approved the final version of the article.

## Details of ethics approval

Natsal‐2 was approved by the University College London and North Thames Multi‐centre research ethics committees and all the local research ethics committees in Britain. The Natsal‐3 study was approved by the Oxfordshire research ethics committee A (ref. no. 09/H0604/27).

## Funding

Natsal‐3 is a collaboration between University College London (London, UK), the London School of Hygiene and Tropical Medicine (London, UK), NatCen Social Research, Public Health England (formerly the Health Protection Agency), and the University of Manchester (Manchester, UK). The study was supported by grants from the Medical Research Council (G0701757) and the Wellcome Trust (084840), with contributions from the Economic and Social Research Council and Department of Health. The sponsors of the study had no role in the study design, data collection, data analysis, data interpretation, or writing of the report. The corresponding author had full access to all of the data in the study and had final responsibility for the decision to submit for publication.
